# Switching costs in stochastic environments drive the emergence of matching behaviour in animal decision-making through the promotion of reward learning strategies

**DOI:** 10.1038/s41598-021-02979-5

**Published:** 2021-12-08

**Authors:** Nan Lyu, Yunbiao Hu, Jiahua Zhang, Huw Lloyd, Yue-Hua Sun, Yi Tao

**Affiliations:** 1 Ministry of Education Key Laboratory for Biodiversity and Ecological Engineering, College of Life Sciences, Beijing Normal University, Beijing, China; 2 Key Laboratory of Animal Ecology and Conservation Biology, Institute of Zoology, Chinese Academy of Sciences, Beijing, People’s Republic of China; 3 Department of Natural Sciences, Faculty of Science and Engineering, Manchester Metropolitan University, Manchester, UK

**Keywords:** Evolution, Neuroscience, Zoology

## Abstract

A principle of choice in animal decision-making named probability matching (PM) has long been detected in animals, and can arise from different decision-making strategies. Little is known about how environmental stochasticity may influence the switching time of these different decision-making strategies. Here we address this problem using a combination of behavioral and theoretical approaches, and show, that although a simple Win-Stay-Loss-Shift (WSLS) strategy can generate PM in binary-choice tasks theoretically, budgerigars (*Melopsittacus undulates*) actually apply a range of sub-tactics more often when they are expected to make more accurate decisions. Surprisingly, budgerigars did not get more rewards than would be predicted when adopting a WSLS strategy, and their decisions also exhibited PM. Instead, budgerigars followed a learning strategy based on reward history, which potentially benefits individuals indirectly from paying lower switching costs. Furthermore, our data suggest that more stochastic environments may promote reward learning through significantly less switching. We suggest that switching costs driven by the stochasticity of an environmental niche can potentially represent an important selection pressure associated with decision-making that may play a key role in driving the evolution of complex cognition in animals.

In response to the uncertainty of natural environments, animals seem to be quite ‘smart’ in making decisions among various options by which they can accrue their fitness efficiently^1,2^. Although the fitness consequences of different decision-making strategies have been the focus of numerous studies, few have examined the animals’ responses to uncertainty and the conditions under which the adoption of or switch to a particular strategy become advantageous^3^.

A general principle of choice in decision-making called probability matching (PM)^4^ has long been identified in animals, including humans^5^. PM occurs when decision-makers match their choice probabilities to a corresponding outcome probability (matching) rather than always choosing the outcome with the highest probability (maximizing)^6,7^. As a result, PM behavior is viewed by many as a ‘suboptimal’ or even an ‘irrational’ strategy^8,9^ because of the comparatively lower expected success rate than that of maximizing (see Supplementary Information 1). Some argue however, that adopting PM can be ‘ecologically rational’ if animals’ regularly encounter a situation in stochastic environments where PM is sufficient for reaching an immediate or short-term goal^13^. Helping to resolve this debate requires a combined theoretical and empirical assessment of why animals adopt non-maximizing behavior, but also identifying the conditions under which PM becomes beneficial in highly stochastic environments.

Psychologists and economists have developed a range of theoretical models for modeling decision-making processes^6,10,11^. Win-Stay-Lose-Shift (WSLS) models have been extensively used to model behavior in decision-making tasks, especially from binary choice experiments^12,13^. In the most basic WSLS model, individuals repeat selections if they succeeded in getting rewards in the last trial (representing a ‘win’), but switch if they failed (a ‘loss’)^13,14^. PM can arise from a WSLS strategy when individuals initially search for patterns by repeat predictions but then change following failures^9^ (see Supplementary Information 1). Consequently, some view PM as simply a byproduct of a local decision-making process^15^ i.e. the outcome of a more complex search for patterns, rather than a strategy per se^9^. PM may also arise from reward (reinforcement) learning, when individuals respond according to an assessment of relatively long historical outcome information^7^. However, reward learning is cognitively more demanding than adopting a simple WSLS strategy, which has been labelled by some as a lazy cognitive shortcut^16^.

Neither are these strategies mutually exclusive as animals may switch between alternative choices, or from one strategy to another. Switching may entail costs for decision-makers, arising primarily from economic considerations^17^. In nature, various switching costs also exist during animal decision making, including not only the energetic and temporal costs during switching^18^, but also costs such as increased predation risk^19^ or that of searching and assessing a new site to improve local familiarity^20^. Although a number of studies have considered such costs in decision-making, little is known about how environmental stochasticity may influence the switching time of different strategies, and then potentially drive the evolution of different decision-making strategies.

Here we bridge those knowledge gaps, using a combination of behavioral experiments and simulation models to examine the use of PM behavior in animal decision-making from an adaptive viewpoint. We firstly use a series of binary choice experiments and theoretical models to investigate the decision-making behavior in budgerigars (*Melopsittacus undulates*), and to determine the role of environmental variability (‘uncertainty’) in driving the use of two different decision-making strategies: WSLS and reward learning. Budgerigars are native to the arid interior habitats of Australia^21^, and are subject to significant spatial and temporal variation in food availability^22^, and consequently they face significant decision-making tasks while searching for rare and patchily distributed food and water sources. Thus, budgerigars are an appropriate species with which to conduct the experiments in this study. Additionally, in order to identify the conditions under which PM behavior can happen and to explore how the more complexed learning strategy would become profitable and adaptive, we construct simulation models based on the budgerigar experimental results.

## Materials and methods

### Binary choice experiments

To test whether animals would really adopt a simple WSLS strategy and exhibit PM behavior, we conducted binary-choice experiments using budgerigars, which have been widely used in studies of different cognitive abilities, such as vocalization learning^23,24^, and problem solving^25,26^. In this study, eighteen unrelated budgerigars were used for the binary-choice experiments and their age ranged from under 1-year-old to 3 years old.

Budgerigars were housed separately in different cages at a size of 20 × 20 × 20 cm prior to each experiment. Binary-choice experiments were conducted in a wire-meshed cage measuring 2 × 1 × 2 m (Supplementary Fig. S1). A single perch was positioned in the center of the cage at a height of 0.8 m from the ground. Two food cups were set on the front wall at a height of 1.6 m from the ground, separated by 1.6 m but only one cup contained the food reward in each trial. For illustration, we denote the side with a higher probability of having rewards as the H-side, and the other side as the L-side in the following. We assume the food rewards would occur on the H-side with a probability *q*, and on the L-side with a probability 1 − *q*.

We first generated sequences of food reward locations for 100 trials under three different random levels (*q* = 0.5, 0.6 and 0.75) using MATLAB (version 7.5, R2007b, The MathWorks Inc.). Each bird was placed in the experimental cage for two days to adapt to the environment, and foraged on food provisioned in the cups in prior (both cups contain foods during this period). Before the experiments, each bird was food deprived for 24 h. Following this, for each experimental trial, we placed approximately 20 grains of millet in the food cup. Once a bird had made a decision and had eaten some millet (after ~ 8–10 s), we removed both food cups, after which the bird would fly back to the perch and wait for the next trial, which was conducted after a period of one minute. If the bird chose a wrong side (i.e., without food rewarding), we would allow it to fly to the other side, after which we immediately removed both food cups from the cage. Since the study subject would become satiated following approximately 30 trails, the total of 100 trials were subsequently conducted over three consecutive days. On each day after conducting the experiments, the bird would be food deprived until the experiments resumed on the following day. To avoid memory interference between random levels, we assigned each bird to only one set of 100 trials. We used three different birds for the experiments under each of the random levels of $q=0.6$ and 0.75, and five birds under the random level of $q=0.5$. To avoid possible effects of side preference, we also used another three different birds for the experiments under each of the random levels of $q=0.6$ and 0.75, and one bird under the random level of $q=0.5$ with the same sequences of food locations, but changing the position of food reward to the opposite side in each trial.

This study complies with all applicable governmental regulations concerning the ethical treatment of animals. All animal use and care was done in compliance with the guidelines of Institute of Zoology, Chinese Academy of Sciences (CAS). This work was permitted by the Animal Care and Use Committee of the Institute of Zoology, CAS.

### Assessing the outcome information for decision-making

To assess how our budgerigars made their decisions through reward learning, we firstly used a one-parameter (time constants $\tau$) leaky integration model to quantify the outcome information in each trial^27^. This model uses a function similar to an exponential filter, which has been derived from a signal processing method^28^. Since food reward was the only income earned by budgerigars during the binary choice experiments, we integrated the reward history of each side as the outcome information. Due to memory capacity limitation^29^ only a finite number of past trials might be informative to decision-makers. Specifically, the outcome information of each side (${y}_{i}={y}_{H}$ for the H-side or ${y}_{L}$ for the L-side) in trial $t$ was calculated as:

${y}_{i}(t) = (1-a){y}_{i}(t-1)+a{x}_{i}(t-1)$ or rewritten as

1 $${y}_{i}\left(t\right)=a\sum_{k=2}^{t}{x}_{i}\left(k-1\right){\left(1-a\right)}^{t-k},$$

where ${x}_{i}(t-1)$ is the income earned in the last one trial (1 or 0), and $a=1-exp\left(-1/\tau \right)$ is a constant between 0 and 1, where $\tau$ is the time constant. We can see that the more recent reward is more informative for making the current decision (Supplementary Fig. S2). Moreover, the reward information from the past $\tau$ trial(s) can explain 63.2% of the output ${y}_{i}(t)$, and the past $3\tau$ and $4\tau$ trials can explain 95% and 98.2% of the output value, respectively.

### Reward learning strategy assessment

To explore how budgerigars made decisions according to the outcome information integrated using different time constants $\tau$, we constructed several generalized linear mixed-effect models (GLMMs) with binomial error (and logit link function) under different time constants $\tau$. In each model, we set the selected side (1 for H-side, and 0 for L-side) in each trial as the dependent variable. The difference in outcome information between the two sides ($\Delta y\left(t, \tau \right)={y}_{H}\left(t, \tau \right)-{y}_{L}\left(t, \tau \right)$) and the random level that individuals encountered (i.e., *q* = 0.5, 0.6 or 0.75) were used as the independent variables in each model. Individual ID was set as a random effect. Normally, as our budgerigars had no prior information to identify different random levels, we would expect random level to be an insignificant factor in the model. Hence, we subsequently assessed the significance of random level in different GLMMs using likelihood ratio tests (LRT) using R function *anova*. The two models used here are shown by the following,

Model 1: Selected side ~ $\Delta y\left(t, \tau \right)$  + random level + (1|ID),

Model 2: Selected side ~ $\Delta y\left(t, \tau \right)$  + (1|ID).

All models were compared using Akaike’s information criterion, AIC^30^, to identify the best-fit time constant in modeling budgerigars’ decisions. Note that we had conducted exploratory analyses by including the side effect (set as 1 or 2 to indicate the experiments conducted under the same sequences with opposite food locations) as another independent variable, which showed that our budgerigars did not have certain side preference during decision-making (see Supplementary Table S1). Hence, the side effect was not considered for further analysis. We had also constructed another three outcome information processing models to assess the decision making of our budgerigars; 1: memory without decay; 2: memory without decay and losing represents a negative income; 3: memory with decay and losing represents a negative income. All of these models showed much higher AIC values than the model described above (see Supplementary Tables S2 and S3). All GLMMs were implemented using function *glmer* in the lme4 package^31^ in R v.3.5.0^32^.

### Simulations of the best-fit statistical model

To determine the robustness of our experimental results and explore how environmental stochasticity influences switching time between decision-making strategies, we conducted computer simulations under different random levels ($q$ ranged from 0.50 to 0.85, stepped by 0.05) to assess the behavior of the deduced reward learning strategy (i.e., the best-fit regression model, see Supplementary Fig. S3); specifically, the choosing probability of the H-side would be predicted using the statistical model in each trial.

For each simulation, we first deduced a reward learning strategy from the best-fit regression model (Supplementary Fig. S3). To capture the uncertainty, we assumed a multivariate normal distribution for regression coefficients. We generated the coefficients of a model of reward learning strategy using the *mvrnorm* function of MASS package in R^33^, with the estimated coefficients of the regression model acting as means and the variance–covariance matrix of different coefficients acting as the variance–covariance matrix for the multivariate normal distribution. In each trial, we calculated the outcome information following the leaky integration model (see Eq. 1), and then passed the difference in outcome information between the two sides to the deduced rewarding learning model to generate the selection probability of the H-side. We ran each simulation for 100 trials and 1000 times under different random levels ($q$ ranged from 0.50 to 0.85, stepped by 0.05). We compared the efficiencies (i.e., success rates) of the model behavior (best-fit regression model) and WSLS strategy, and verified whether the model behavior could reduce the number of switching events efficiently.

## Results

### Modelling and testing the adoption of WSLS strategy in budgerigars

**Figure 1:**
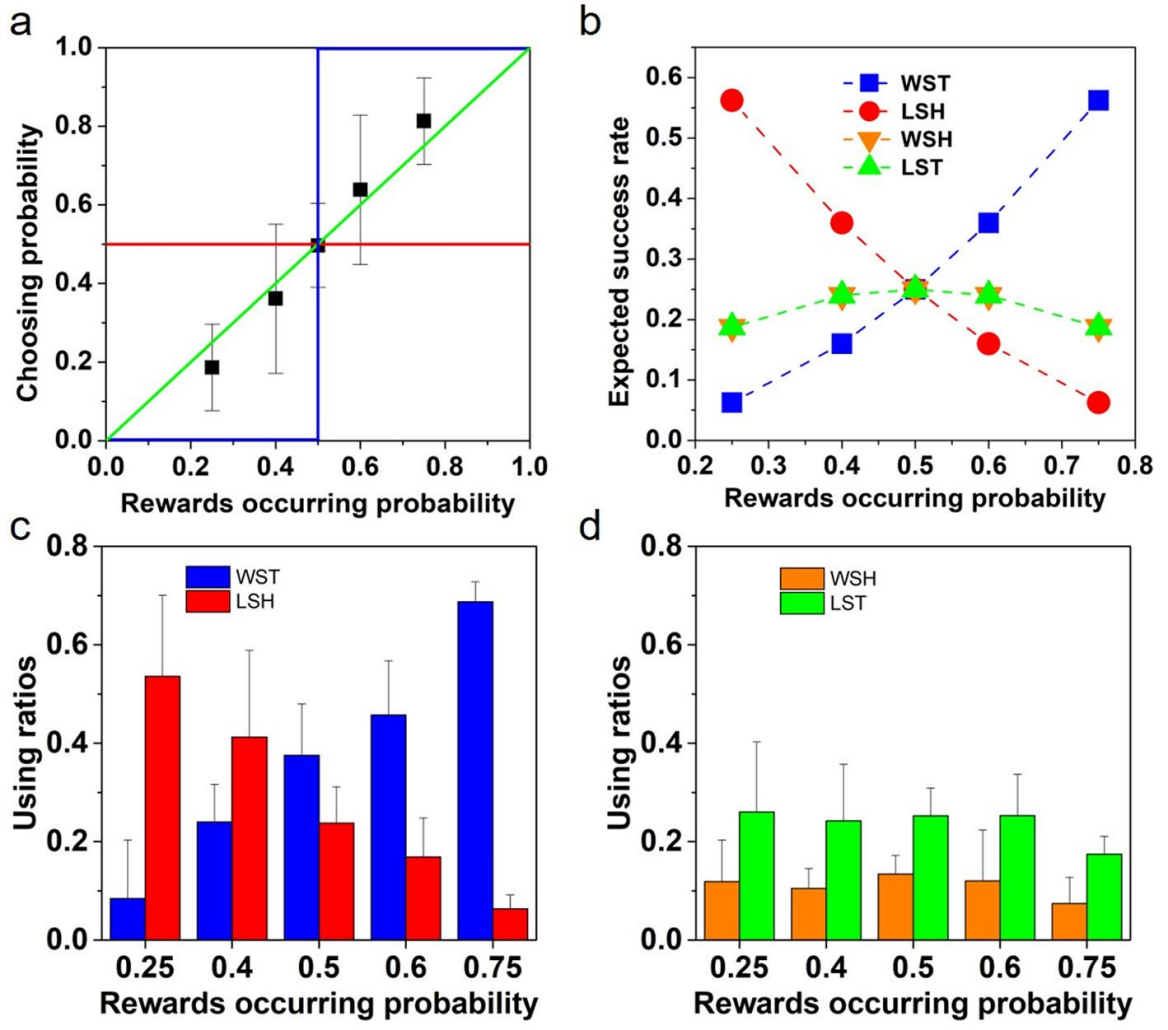
Decision-making by budgerigars under different binary choice experimental conditions. (**A**) Choosing probabilities of the side with different food occurrence probabilities. The black squares with bars show the mean (± SD) choosing probabilities of budgerigars in the binary choice experiments. The expected choosing probabilities using the maximizing, WSLS and random strategy are shown by the blue, green and red lines, respectively. (**B**) The expected accuracy rates of the four sub-tactics (i.e., WST, LSH, WSH and LST) under different food occurrence probabilities. (**C**) and (**D**) show the mean (± SD) relative use ratios of each sub-tactic (i.e., WST, LSH, WSH and LST) in decision-making by our budgerigars.

**Figure 2:**
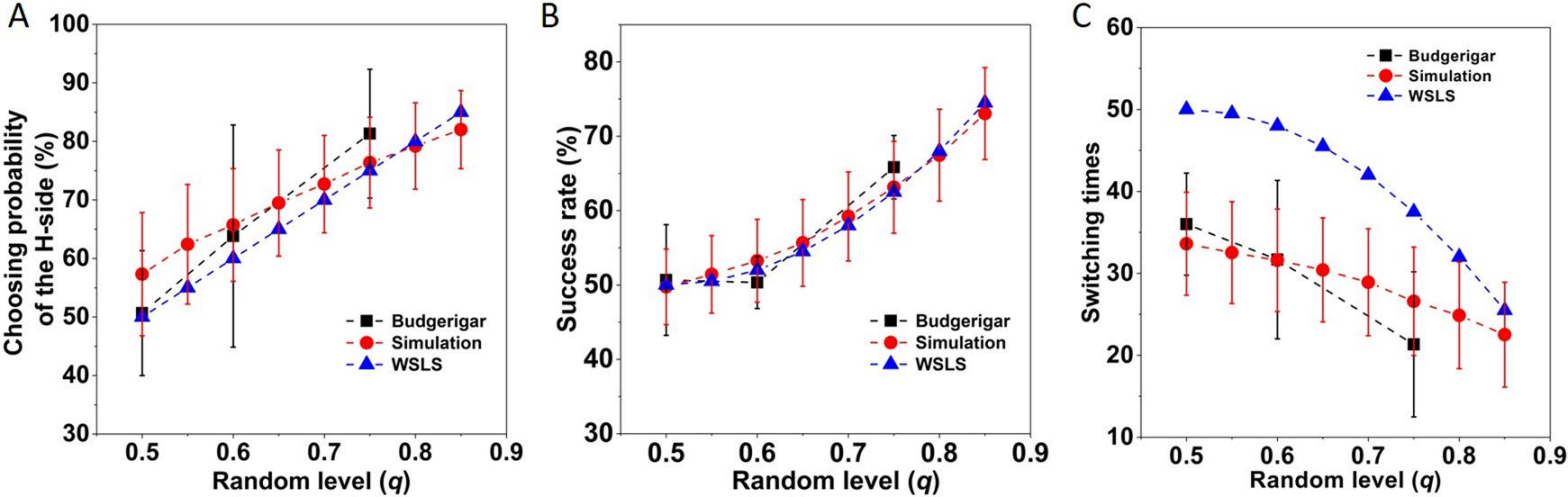
Choosing probability of the H-side (**A**), success rate (**B**) and mean switching times (**C**) of budgerigars (black squares with SD bars) and the simulated results (red circles with SD bars) using the best-fit statistical model in 100 trials. The blue triangles represent the expected results when decision-makers adopt the WSLS strategy.

Our results identified PM by budgerigars (Fig. 1A); however, they did not adopt a WSLS strategy as expected. Specifically, when the food rewards probability increased, the relative frequency of using the win-stay (WST) sub-tactic would increase, while the relative frequency of using a lose-shift (LSH) sub-tactic would decrease (Fig. 1C). The relative frequencies of using lose-stay (LST) and win-shift (WSH) sub-tactics were stable under different food rewards probabilities (Fig. 1D). Interestingly, the corresponding expected accuracy of each sub-tactic (i.e., WST, LSH, LST, WSH) showed a similar pattern to the relative use frequency (Fig. 1B). Thus, our budgerigars were able to apply the more accurate sub-tactics more often for their decision-making. Nonetheless, neither the choosing probability of each side (Fig. 1A) nor the mean accuracies in getting rewards differed from adopting the simple WSLS strategy (Fig. 2A,B). How does this seemingly contradictory phenomenon arise?

### Modelling reward learning in budgerigar decision-making

We now consider the reward learning strategy in modeling our budgerigars’ decisions. Likelihood analyses indicated that random level showed a relatively significant effect until the time constant $\tau$ increased to two (Table 1). Furthermore, when $\tau =1$, excluding the random level would result in a model with a much higher AIC value (with $\Delta AIC=3.8$), representing a poorly supported model, based on the conventional rule of thumb in model selection ($\Delta AIC<2$, see Ref.^34^). Therefore, our budgerigars should follow a memory integration model with a time constant $\tau$ of at least two when undertaking binary choice tasks. We also found that the model constructed under an even larger time constant $\tau$ ($>2$) would have a higher AIC value (with $\Delta AIC>2$; Table 1); thus the GLMM constructed under $\tau =2$ showed the best-fit for modeling decision-making in our budgerigars. These findings suggest that our budgerigars are more likely to make their decisions according to a relatively long history of outcome information (rather than using only one previous trial in WSLS), raising new questions about why this comparatively more complex learning mechanism could potentially evolve without additional benefits as we detected in our budgerigars (Fig. 2B).

**Table 1:** Generalized linear mixed models (GLMMs) constructed to analyze the effects of difference in outcome information (Δy) and random level (0.5, 0.6 or 0.75) under different time constants (τ). ΔAIC is calculated as the AIC value of the model excluding the variable of random level minus that of the model with the random level. χ^2^ and P values represent the likelihood analyses results (i.e., comparing models with versus without the variable of random level using R function *anova*).

Time constant (τ)	AIC (with random level)	AIC (without random level)	ΔAIC	χ^2^	P value
1	1872.6	1876.4	3.8	5.826	0.016*
2	1870.9	1872.2	1.3	3.304	0.069
3	1884.3	1884	− 0.3	1.723	0.189
4	1898.9	1897.7	− 1.2	0.808	0.369
5	1912.5	1910.8	− 1.7	0.335	0.563
6	1924.6	1922.7	− 1.9	0.119	0.730
7	1935.4	1933.4	− 2	0.035	0.853
8	1945	1943	− 2	0.007	0.932
9	1953.6	1951.6	− 2	0.001	0.975
10	1961.3	1959.4	− 1.9	< 0.001	0.989

### Lower switching costs of reward learning strategy

Through conducting simulations, we firstly confirmed that using a reward learning strategy would cause an increased use ratio of WST and a decreased use ratio of LSH when the food rewards occurring probability increased (Supplementary Fig. S4), as we had detected in our budgerigars (see Fig. 1C,D). Furthermore, compared to the simple WSLS strategy, reward learning did not cause decision-makers to select the H-side much more often (Fig. 2A) or to acquire a higher success rate (Fig. 2B). However, reward learning did result in much less switching especially when food rewards were more evenly distributed between the two sides (i.e., when *q* is getting closer to 0.5, Fig. 2C). Therefore, the reward learning strategy should be increasingly less costly for decision-makers than the WSLS strategy under more variable environments.

## Discussion

Although PM can occur by adopting the simple WSLS strategy, we found that our budgerigars were more likely to adopt the comparatively more complex reward learning. Complex cognition has long been suggested as an adaptation to environmental stochasticity^35^. However, evidence from studies that examined relatively larger-brained birds exposed to more variable environments (e.g. Ref.^36^) and which have examined how environmental enrichment can promote the cognitive ability in fishes (e.g. Ref.^37^) did not provide any direct causation mechanisms.

Using a combination of behavioral, theoretical and computational approaches, we illustrate that the more complex reward learning actually cannot outperform a WSLS strategy through gaining more rewards, but can potentially benefit individuals indirectly from paying lower switching costs. Furthermore, environmental variability plays a fundamental role in determining the switching time of each strategy, and the more variable environments may promote the evolution of reward learning through significantly less switching. In primates, PM represents an adaptive strategy for foraging in stochastic environments, driven by reward learning^38^. Experimental studies have suggested that evolved reward learning is sufficient for PM in bees^39^, particularly in situations that require simultaneous sampling of different individual flowers of the same or different species, while harvesting from the best estimated flower type in a patch^40–42^. Additionally, foraging bees have evolved to use only a subset of decision-making strategies that are most adaptive to environmental stochasticity, as this allows bees to track variation in both the quality and availability of food sources^40^. Thus, PM may represent an ecologically optimal foraging solution for animals such as budgerigars if reward learning probabilities are highly variable^43^, whilst also evolving in less competitive environments for the birds as a direct result of near-optimal reward learning^39^.

Some argue that PM can evolve when environmental stochasticity is systematic across all individuals i.e., that natural selection is able to yield behaviours that may be individually sub-optimal but are optimal for the population^44^. As a native species from Australia, budgerigar populations are known for their nomadic ecology, which is tied to significant spatial and temporal variation in food and water availability over vast arid landscapes^21,22^. This species tends to occur in small flocks but can form significantly larger flocks when environmental conditions worsen, such as during periods of drought^45^. Consequently, they face significant decision-making tasks while searching for food and water sources. Since our data suggest that budgerigars would use reward learning strategy that may potentially permit the evolution of PM, this suggests that most foraging situations they encounter in the wild (e.g., depletion of food resources via intra-specific competition, drought-dependent variation in seed production) favour a more limited range of decision-making strategies, as has been suggested for bees^40^. In these uncertain arid environments, frequent switching among foraging sites would not enable budgerigars to get more food, while flying during such hot and dry environmental conditions would consume a lot of energy and water. Thus, developing the capability of integrating outcome information from a longer history of rewards, though cognitively more complex, might be relatively more cost-efficient to budgerigars in the wild. We therefore draw attention to a potentially important selection pressure associated with decision-making (i.e., switching cost) that may play a key role in driving the evolution of complex cognition in animals.

In this study, we constructed leaky integration models with different time constants ($\tau$) to assess the reward learning processes of captive budgerigars. Generally, a higher value of the constant $\tau$ would give rise to more sluggish responses to changes in the outcome^27^, because decision-makers would integrate the outcome information from a longer history of rewards. In binary-choice experiments, although the H-side is more likely to have rewards than the L-side in each trial, the reward can still occur on the L-side more often than on the H-side within *n* (> 1) trials, representing a mismatching situation. Theoretically, an increasing of the trial number *n* can effectively reduce the mismatching rate $\rho$ (see Supplementary Fig. S5), and therefore, integrating the outcome from a longer history can potentially enable the decision-makers to identify the two sides more accurately. On the other hand, a lower mismatching rate also implies that the ratio of switching between the matching and mismatching situations should be lower, which may in turn reduce the intention of shifting between the two sides by decision-makers. Specifically, as shown with our budgerigars, a leaky integration model with the time constant $\tau =2$ showed the best-fit in modeling their decisions, by which they can have much less switching than the WSLS strategy when executing binary-choice tasks (Fig. 2C).

It is important to note that we make no claim that our deduced statistical model exactly captures our budgerigars’ decisions. The model is just for descriptive purpose. While conducting the binary choice tasks, the only information that could be used by the birds are memories regarding previous trials (e.g., the side they selected and whether they successfully obtained the rewards). For stochastic environments, animals should only consider a small number of recent experiences (the WSLS strategy considers the memory of one previous trial, while the reward learning strategy considers memories from more than one previous trial) since older experiences may not be as informative about the current situation^29,38^—a factor specified in our leaky integration models. As it can be seen that comparing to the other three sets of GLMMs (see Supplementary Tables S2 and S3), those ones constructed using the outcome information assessed through a leaky model generally showed better-fitting (Table 1). Nonetheless, we found that even using the best-fit regression model to conduct simulations still did not perfectly describe budgerigar behaviour (see Fig. 2). It thus may imply that other sub-strategies might be employed. Future studies should consider this and whether budgerigars can adjust their learning rate and switching as environmental stochasticity changes on multiple timescales.

In nature, animals are faced with variation in environmental conditions, and there are also variations in the switching costs during decision-making. We suggest that these may in turn drive the evolution of species-specific memory processing and other cognitive capabilities. Specifically, those species that live in more variable environments and/or have higher switching costs should prefer less switching and thus might be more likely to evolve the more complex cognition in general.

## Supplementary Information

Supplementary Information.
