# Supplementary material for: Switching costs in stochastic environments drive the emergence of matching behaviour in animal decision-making through the promotion of reward learning strategies
Source: Sci Rep. 2021 Dec 8;11:23593. doi: 10.1038/s41598-021-02979-5 (PMC8654859; doi:10.1038/s41598-021-02979-5)
Supplement: Supplementary file 1 — Supplementary Information. [file 41598_2021_2979_MOESM1_ESM.docx]

**Supplementary materials for**

**Switching costs in stochastic environments drive the emergence of matching behaviour in animal decision-making through the promotion of reward learning strategies**

Nan Lyu^1,2,^^*^, Yunbiao Hu^2^, Jiahua Zhang^2^, Huw Lloyd^3^, Yue-Hua Sun^2,*^ and Yi Tao^2,*^

^1^ Ministry of Education Key Laboratory for Biodiversity and Ecological Engineering, College of Life Sciences, Beijing Normal University, Beijing, China

^2^ Key Laboratory of Animal Ecology and Conservation Biology, Institute of Zoology, Chinese Academy of Sciences, Beijing, People's Republic of China

^3^ Department of Natural Sciences, Faculty of Science and Engineering, Manchester Metropolitan University, Manchester, United Kingdom

^*^ email: nanlyu@bnu.edu.cn, sunyh@ioz.ac.cn, yitao@ioz.ac.cn

**Running Title:** Matching behavior in animal decision-making

**Text 1. Modeling the WSLS, random and maximizing strategy**

In the WSLS model, decision-makers just repeat selections if they succeeded in the previous trial, but switch if they failed. Let *q* denote the probability of the food rewards occurring on the H-side, and then 1-*q* is the probability of the other side. Let *x* denote the choosing probability of the H-side, and then 1-*x* is the selection probability of the other side. While using the WSLS strategy, decision-makers will select the H-side when it wins on this side (i.e., with a probability *qx*) or loses on the other side (i.e., with a probability *q*(1-*x*)). Therefore, the selection probability of the H-side is equal to $qx+q\left( 1-x \right)$, i.e., $x=q$. Similarly, we can deduce that the choosing probability of the other side is equal to $1-q$. This implies that PM can definitely occur when individuals make their decisions simply considering only one previous trial and use the WSLS strategy.

The success rate of the WSLS strategy is $q^{2}+{(1-q)}^{2}$, which is always larger than $0.5$ if$q>0.5$. Note that if decision-makers just choose the side randomly during each trial (see the red line in Fig. 1a in the main text) they can only succeed at a rate of 0.5 under any random level. The best maximizing strategy should be choosing the H-side in each trial, which can enable the bird to get food rewards at a rate of $q$ (when $q\geq0.5$) or $1-q$ (when $q<0.5$) (see the blue line in Fig. 1a in the main text).

Table S1. Results of the exploratory GLMM analyzing the effects of difference in outcome information (Δy), random level (0.5, 0.6 or 0.75) and side effect (set as 1 or 2 to indicate the experiments conducted under the same sequences with opposite food locations).

| Time constant (𝜏) | Δy |  | Random level |  | side effect |  | AIC | ΔAIC^*^ | P value^*^ |
| --- | --- | --- | --- | --- | --- | --- | --- | --- | --- |
|  | z | P | z | P | z | P |  |  |  |
| 1 | 13.528 | <0.001 | 2.264 | 0.024 | 1 | 0.3175 | 1873.6 | -1 | 0.323 |
| 2 | 13.467 | <0.001 | 1.554 | 0.12 | 1.004 | 0.315 | 1872 | -1.1 | 0.321 |
| 3 | 13.009 | <0.001 | 1.027 | 0.304 | 0.996 | 0.319 | 1885.4 | -1 | 0.324 |
| 4 | 12.516 | <0.001 | 0.629 | 0.53 | 0.985 | 0.325 | 1900 | -1.1 | 0.329 |
| 5 | 12.057 | <0.001 | 0.33 | 0.741 | 0.971 | 0.331 | 1913.5 | -1 | 0.335 |
| 6 | 11.649 | <0.001 | 0.115 | 0.908 | 0.956 | 0.339 | 1925.7 | -1.1 | 0.342 |
| 7 | 11.286 | <0.001 | -0.031 | 0.975 | 0.939 | 0.348 | 1936.5 | -1.1 | 0.350 |
| 8 | 10.964 | <0.001 | -0.121 | 0.904 | 0.922 | 0.356 | 1946.2 | -1.2 | 0.358 |
| 9 | 10.674 | <0.001 | -0.166 | 0.868 | 0.906 | 0.365 | 1954.8 | -1.2 | 0.366 |
| 10 | 10.41 | <0.001 | -0.176 | 0.86 | 0.891 | 0.373 | 1962.6 | -1.2 | 0.374 |

* Changes in AIC values (ΔAIC) showed the AIC value of the model without the variable of side effect minus that of the model with the side effect; P-values was obtained through LRT analysis, i.e., by comparing models with versus without the variable of side effect.

Table S2. Generalized linear mixed models (GLMMs) constructed to analyze the effects of difference in outcome information (Δy’) and random level (0.5, 0.6 or 0.75) under different periods (*T*) without memory discount or decay. ΔAIC is calculated as the AIC value of the model excluding the variable of random level minus that of the model with the random level. χ2 and P values represent the likelihood analyses results (i.e., comparing models with versus without the variable of random level using R function *anova*).

| Model | Time period (T) | AIC (with random level) | AIC (without random level) | ΔAIC | χ^2^ | P value |
| --- | --- | --- | --- | --- | --- | --- |
| Model A | 2 | 1901.3 | 1906.0 | 4.7 | 6.690 | 0.010* |
|  | 3 | 1905.3 | 1909.0 | 3.7 | 5.688 | 0.017* |
|  | 4 | 1932.5 | 1936.0 | 3.5 | 5.507 | 0.019* |
|  | 5 | 1947.5 | 1950.6 | 3.1 | 5.142 | 0.023* |
|  | 6 | 1956.4 | 1959.1 | 2.7 | 4.720 | 0.030* |
|  | 7 | 1966.2 | 1968.7 | 2.5 | 4.477 | 0.034* |
|  | 8 | 1972.8 | 1975.0 | 2.2 | 4.229 | 0.040* |
|  | 9 | 1975.3 | 1977.2 | 1.9 | 3.917 | 0.048* |
|  | 10 | 1977.8 | 1979.5 | 1.7 | 3.653 | 0.056 |
| Model B | 2 | 1987.2 | 1993.9 | 6.7 | 8.639 | 0.003* |
|  | 3 | 1988.2 | 1993.8 | 5.6 | 7.574 | 0.006* |
|  | 4 | 2003.5 | 2008.6 | 5.1 | 7.121 | 0.008* |
|  | 5 | 2023.9 | 2028.9 | 5 | 7.078 | 0.008* |
|  | 6 | 2033.4 | 2038.3 | 4.9 | 6.879 | 0.009* |
|  | 7 | 2039.2 | 2043.9 | 4.7 | 6.662 | 0.010* |
|  | 8 | 2043.0 | 2047.5 | 4.5 | 6.465 | 0.011* |
|  | 9 | 2035.2 | 2038.9 | 3.7 | 5.666 | 0.017* |
|  | 10 | 2033.6 | 2036.9 | 3.3 | 5.294 | 0.021* |
|  | 11 | 2037.3 | 2040.5 | 3.2 | 5.182 | 0.023* |
|  | 12 | 2038.3 | 2041.2 | 2.9 | 4.940 | 0.026* |
|  | 15 | 2049.0 | 2051.8 | 2.8 | 4.816 | 0.028* |
|  | 20 | 2056.7 | 2059.2 | 2.5 | 4.46 | 0.035* |
|  | 25 | 2056.2 | 2057.6 | 1.4 | 3.453 | 0.063 |
|  | 28 | 2057.6 | 2058.7 | 1.1 | 3.15 | 0.076 |
|  | 30 | 2049.8 | 2049.9 | 0.1 | 2.119 | 0.145 |

In Model A, the outcome information (Δy’) is calculated as the differences of rewards gained between the two sides in *T* previous trials without memory discount or decay (i.e., $\Delta y’(t)=\sum_{k=1}^{T} [x_{H}\left( t-k \right)-x_{L}\left( t-k \right)]$, where $x_{i}\left( t \right)$ represents the income earned in the *t*th trial, and $x_{i}\left( t \right)=1$ when decision-maker got the rewards, otherwise $x_{i}\left( t \right)=0$). In model B, we further assume that choosing a side without gaining rewards would have a negative income, i.e., $x_{i}\left( t \right)=1$ represents the decision-maker got the rewards on the side; $x_{i}\left( t \right)=0$ represents the decision-maker did not choose the side; $x_{i}\left( t \right)=-1$ represents the decision-maker chose the side without getting rewards.

Table S3. Generalized linear mixed models (GLMMs) constructed to analyze the effects of difference in outcome information (Δy’’) and random level (0.5, 0.6 or 0.75) under different time constants (𝜏). In this model, we further assume that choosing a side without gaining rewards would have a negative income. ΔAIC is calculated as the AIC value of the model excluding the variable of random level minus that of the model with the random level. χ2 and P values represent the likelihood analyses results (i.e., comparing models with versus without the variable of random level using R function *anova*).

| Time constant (𝜏) | AIC (with random level) | AIC (without random level) | ΔAIC | χ^2^ | P value |
| --- | --- | --- | --- | --- | --- |
| 1 | 1984.2 | 1989.6 | 5.4 | 7.322 | 0.007* |
| 2 | 1980.8 | 1984.3 | 3.5 | 5.498 | 0.019* |
| 3 | 1986.6 | 1988.9 | 2.3 | 4.334 | 0.037* |
| 4 | 1992.4 | 1993.9 | 1.5 | 3.491 | 0.062 |
| 5 | 1996.9 | 1997.8 | 0.9 | 2.826 | 0.093 |
| 6 | 2000.4 | 2000.7 | 0.3 | 2.281 | 0.131 |
| 7 | 2003.2 | 2003.0 | -0.2 | 1.834 | 0.176 |
| 8 | 2005.4 | 2004.8 | -0.6 | 1.471 | 0.225 |
| 9 | 2007.3 | 2006.5 | -0.8 | 1.180 | 0.277 |
| 10 | 2008.9 | 2007.9 | -1 | 0.9509 | 0.330 |

The outcome information (Δy’’) is calculated using the same leaky integration model (see equation 1 in the main text). However, $x_{i}\left( t \right)$ can have three different values in this case, i.e., $x_{i}\left( t \right)=1$ represents the decision-maker got the rewards on the side; $x_{i}\left( t \right)=0$ represents the decision-maker did not choose the side; $x_{i}\left( t \right)=-1$ represents the decision-maker chose the side without getting rewards.


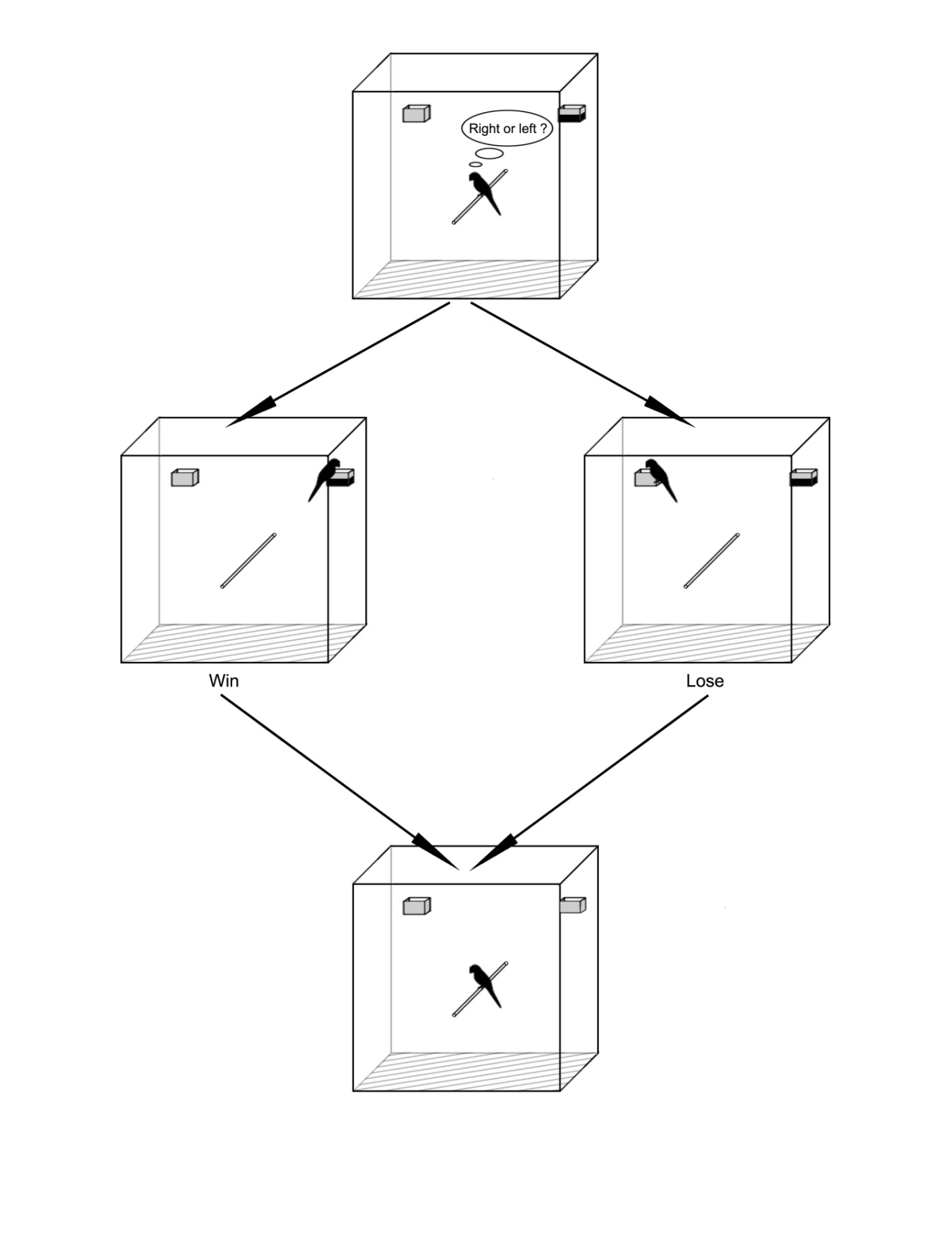


Figure S1. Diagrammatic representation of the experimental apparatus and procedures for testing the adoption of the WSLS strategy in budgerigars.


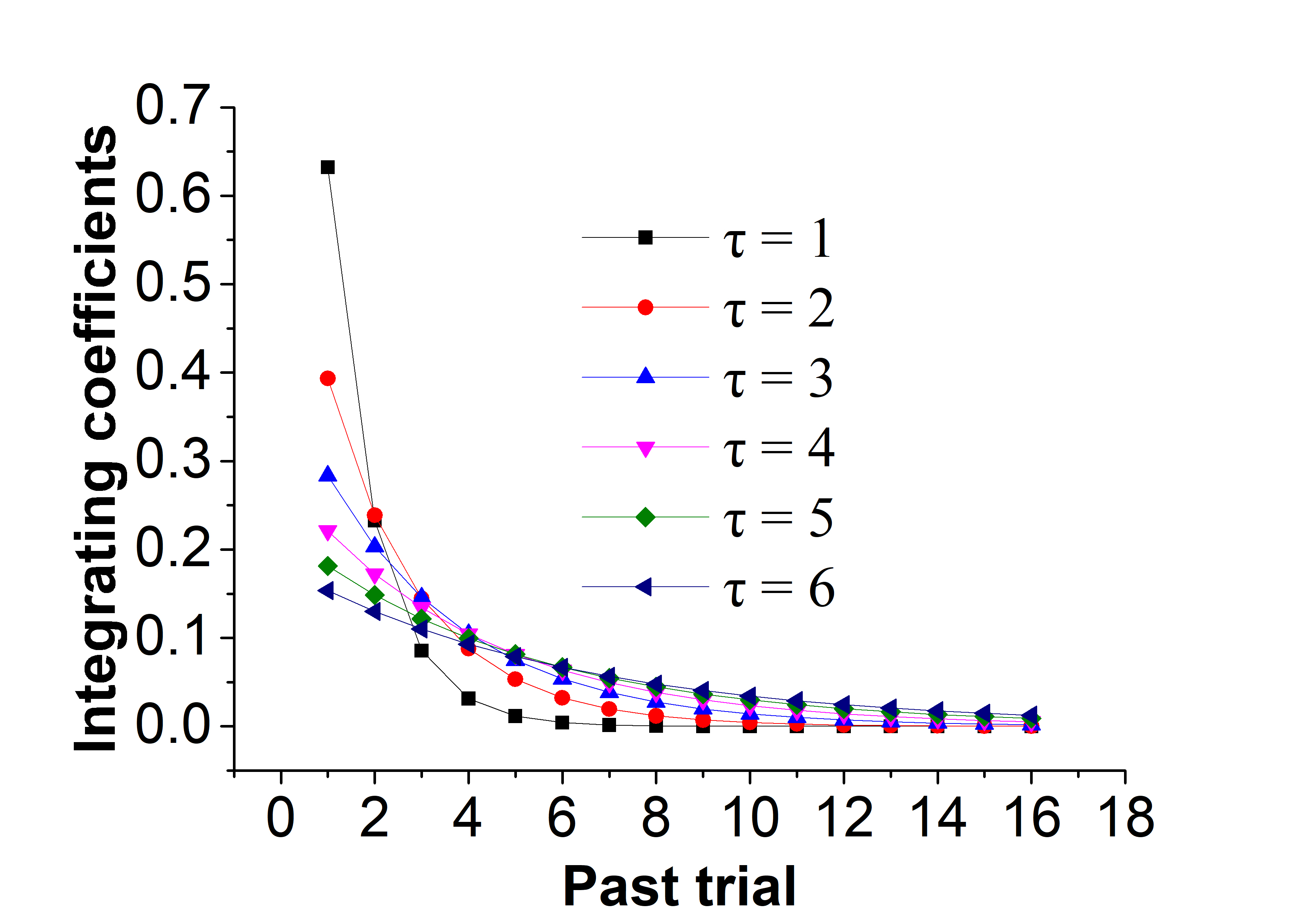


Figure S2. The integrating coefficients of outcome information in the past trials under different time constants (𝜏). The coefficient of the past *i*-th trial was calculated as ${a\left( 1-a \right)}^{i-1}$, where $a=1-exp\left( -1/\tau\right)$.


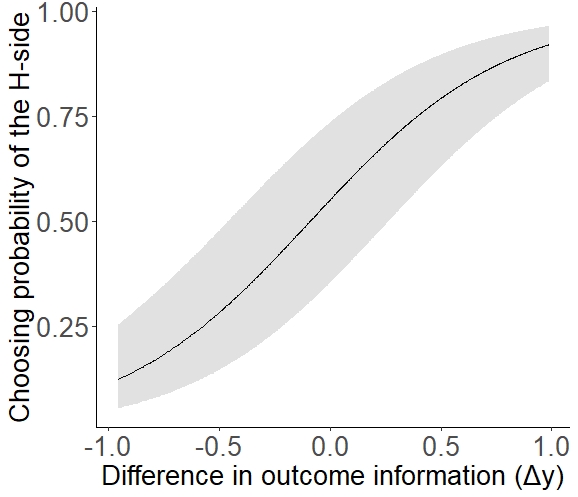


Figure S3. The predicted results of the best-fit GLMM (when 𝜏 = 2) analyzing the effects of difference in outcome information (Δy) on the choosing probability of the H-side. The grey area shows the 95% CI.


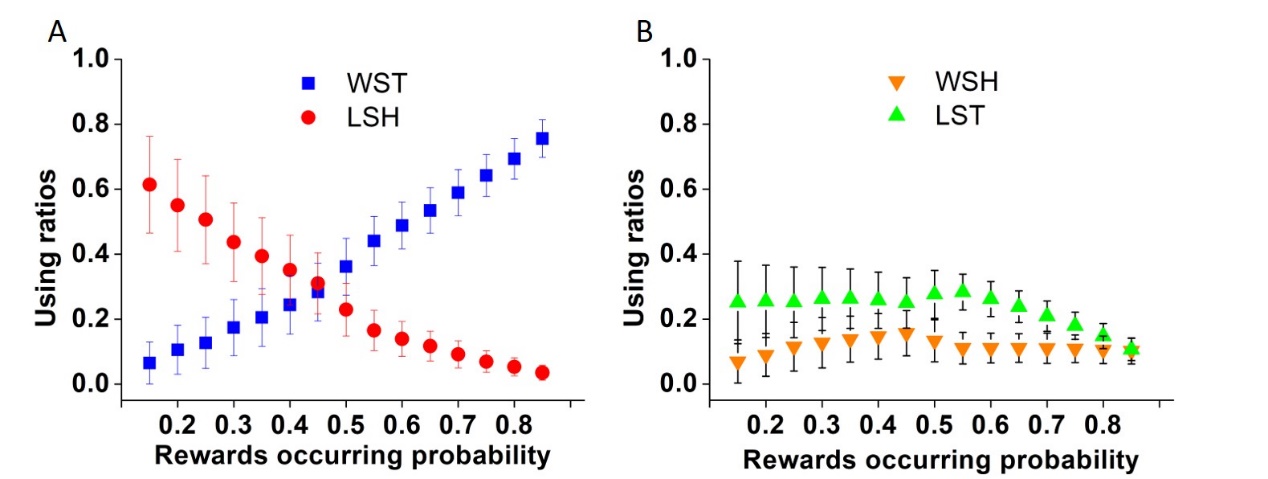


Figure S4. The mean (± SD) relative using ratios of each sub-tactic (i.e., WST, LSH, WSH and LST) in decision-making simulations. We ran simulations for 100 trials and 1000 times under different random levels ($q$ ranged from 0.50 to 0.85, stepped by 0.05).


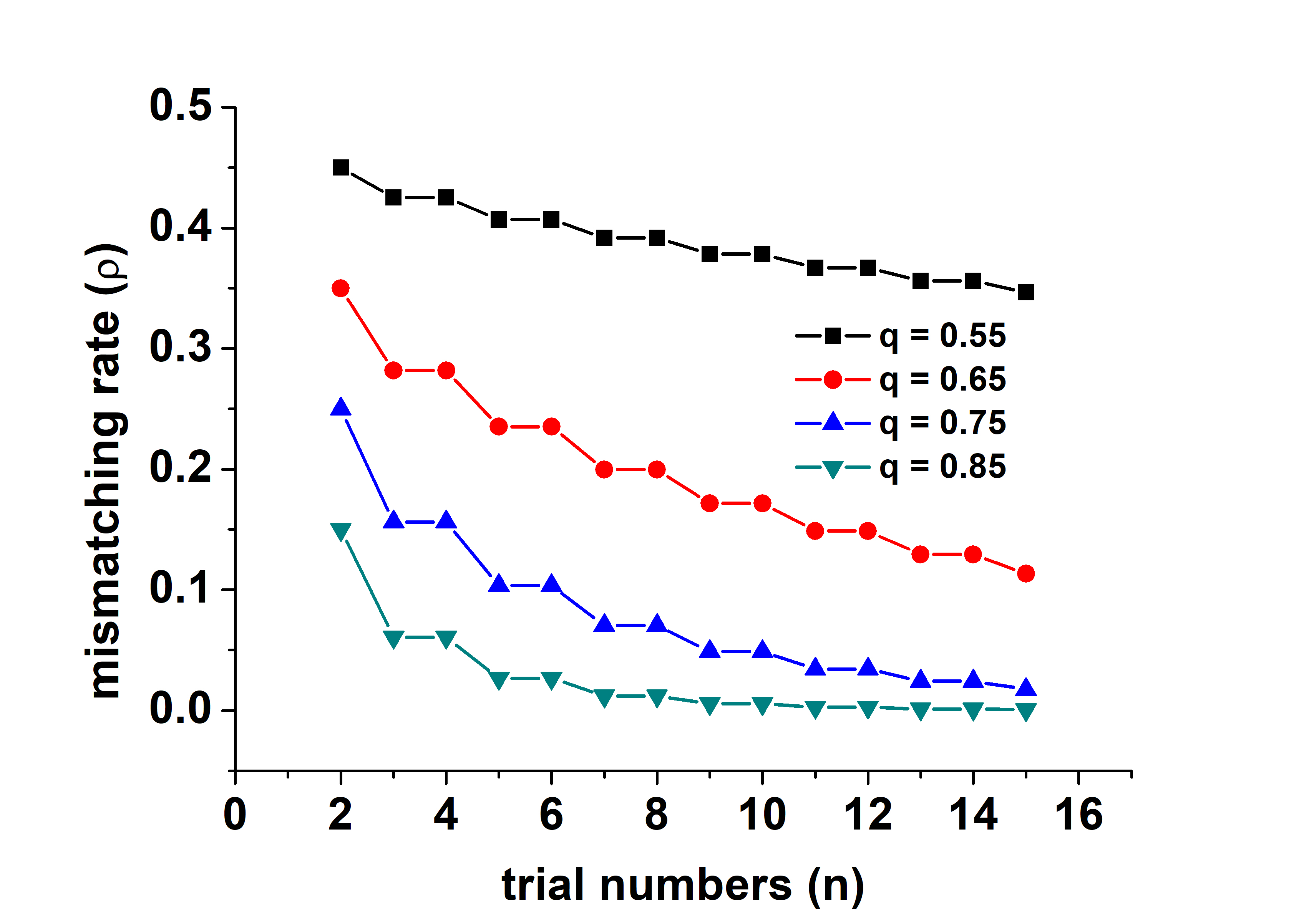


Figure S5. The mismatching rate ($\rho$) under different binary-choice environments (*q* = 0.55, 0.65, 0.75 and 0.85) and trial numbers (*n*). This rate represents the probability of a situation when the reward occurs on the L-side more often than on the H-side within *n* trials, which is calculated as $\sum_{i=0}^{(n-1)/2} C_{n}^{i}q^{i}\left( 1-q \right)^{n-i}$ when *n* is odd, and $\sum_{i=0}^{(n-2)/2} C_{n}^{i}q^{i}\left( 1-q \right)^{n-i}+\frac{1}{2}C_{n}^{n/2}q^{n/2}\left( 1-q \right)^{n/2}$, otherwise.
